# A global-wide search for sexual dimorphism of glomeruli in the antennal lobe of female and male *Helicoverpa armigera*

**DOI:** 10.1038/srep35204

**Published:** 2016-10-11

**Authors:** Xin-Cheng Zhao, Bai-Wei Ma, Bente G. Berg, Gui-Ying Xie, Qing-Bo Tang, Xian-Ru Guo

**Affiliations:** 1 Department of Entomology, College of Plant Protection, Henan Agricultural University, Zhengzhou, 450002, China; 2 Department of Psychology, Norwegian University of Science and Technology, Trondheim 7489, Norway; 3 Collaborative Innovation Center of Henan Grain Crops, Zhengzhou, 450002, China

## Abstract

By using immunostaining and three-dimensional reconstruction, the anatomical organization of the antennal lobe glomeruli of the female cotton bollworm *Helicoverpa armigera* was investigated. Eighty-one glomeruli were identified, 15 of which were not previously discovered. The general anatomical organization of the AL of female is similar to that of male and all glomeruli were classified into four sub-groups, including the female-specific glomerular complex, posterior complex, labial-palp pit organ glomerulus, and ordinary glomeruli. A global-wide comparison on the complete glomerular map of female and male was performed and for the first time the quantitative difference in volume for each individual homologous glomerulus was analyzed. We found that the sexual dimorphism includes not only the sex-specific glomeruli but also some of the other glomeruli. The findings in the present study may provide a reference to examine the antennal-lobe organization more in detail and to identify new glomeruli in other moth species. In addition, the complete identification and global-wide comparison of the sexes provide an important basis for mapping the function of distinct glomeruli and for understanding neural mechanisms underlying sexually dimorphic olfactory behaviors.

The antennal lobe (AL), an analogue to the olfactory bulb in vertebrates, is the primary olfactory center of the insect brain^1^,^2^,^3^,^4^. Odor information processing in the AL is mainly performed by olfactory sensory neurons (OSN), projection neurons, and local interneurons^1^,^2^,^3^. In general, these neurons form dense synaptic contacts in the spheroidal neuropilar subcompartments named glomeruli. Each glomerulus works as a functional unit in olfactory information processing^2^,^3^. In *Drosophila melanogaster*, OSNs expressing the same receptor type are shown to converge onto one or two glomeruli in the primary olfactory center^5^,^6^,^7^. Particular odors are thus represented in distinct glomeruli^7^,^8^. The anatomical organization of glomeruli shows species-, sex-, and caste-specific adaptations in relation to the particular biology and ecology of different species^4^,^9^,^10^,^11^,^12^. In many moths, electrophysiological and imaging recordings combined with anatomical studies have demonstrated that the male-specific glomeruli, the macroglomerular complex (MGC), encode sex pheromone information, whereas a larger number of sexually isomorphic, ordinary glomeruli (OGs), encode host plant volatiles^2^,^3^,^13^,^14^.

Generally, each glomerulus is unique as regards shape, size, and relative location^4^,^15^,^16^. Anatomical identification of individual glomeruli provides the basis for corresponding functional characterization as well as for examining the overall organization of the AL in different species, sexes, and castes. Also, it enables investigations on neural plasticity related to learning mechanisms^17^. Anatomical maps of the identified glomeruli were made in many different insect species, including honey bee^18^,^19^, fruit fly^16^,^20^, parasitoids wasps^21^, mosquitoes^22^,^23^, and Lepidopteran species^24^,^25^,^26^,^27^,^28^,^29^,^30^,^31^,^32^,^33^,^34^ (*Pieris brassicae*^24^ and *Mamestra brassicae*^24^; *Manduca sexta*^25^,^26^; *Heliothis virescens* and *Helicoverpa assulta*^27^; *Agrotis ipsilon*^28^; *Lobesia botrana*^29^; *Helicoverpa armigera*^30^; *Spodoptera littoralis*^31^; *Bombyx mori*^32^; *Cydia molesta*^33^; *H. virescens*^34^).

Recently, the anatomical organization of the AL glomeruli of the male cotton bollworm *Helicoverpa armigera* was investigated more in detail by performing labeling of the AL associated neurons in addition to synaptic antibody staining^35^. The boundaries of the AL and all glomeruli were accurately determined and a substantially larger number of glomeruli, about 80, were identified as compared to that previously reported in *H. armigera* and even in all other studied moths^35^. Fifteen newly discovered glomeruli located in the posterior part of AL, previously considered to be a part of the protocerebrum, were identified.

Based on the new findings, the anatomical organization of the AL glomeruli of the female *H. armigera* was therefore re-investigated. Besides, a global-wide comparison of female and male was performed in order to identify sexually dimorphic and isomorphic glomeruli. Also, we performed quantitative analysis, for the first time, to examine volume differences between homologous glomeruli in female and male. Generally, the size of individual glomeruli is assumed to reflect the number of sensory axons terminating in the neuropil structures. The cumulus of the MGC in *H. armigera* males, for example, which receives input from numerous sensory neurons tuned to the major pheromone component, *cis-*11-hexadecenal, is substantially enlarged as compared to the other glomeruli^30^. These kinds of characteristics, applying to sexually dimorphic glomeruli, obviously concerns distinct sex-specific behaviors. However, isomorphic glomeruli displaying different volumes in the two sexes may also indicate male- versus female-specific responses. The presented data is therefore important not only for future studies aiming at mapping odor qualities to the specific glomeruli but also for investigating the neural basis for sex-specific olfactory behaviors, particularly in females.

## Results

### General anatomy of the antennal lobe of female *H. armigera*

**Figure 1:**
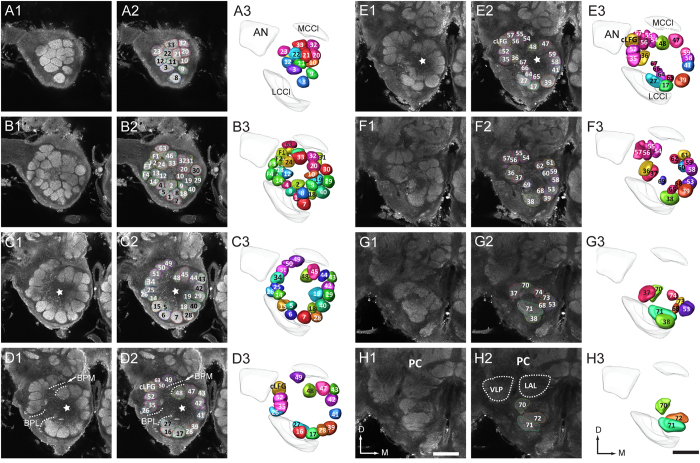
Confocal images and three-dimensional reconstructions of the antennal-lobe (AL) glomeruli in a frontal view. **A1–H1:** confocal images of AL sections at different depths. The most anterior glomeruli, at 27 μm (**A**). 42 μm (**B**). 63 μm (**C**). 78 μm (**D**). 90 μm (**E**). 105μm (**F**). 123 μm (**G**). 138 μm (**H**). **A2–H2:** Same confocal images as shown in **A1**–**H1**, but with labeling. **A3–H3:** Three-dimensional reconstructions of glomeruli based on the confocal images shown in **A2**–**H2** (frontal view). The stars indicate the antennal lobe hub. AN: antennal nerve; cLFG: central large female glomerulus; LAL: lateral accessory lobe; LCCl: lateral cell body cluster; BPL: bundle of primary neurites connected to the LCCl; MCCl: medial cell body cluster; BPM: bundle of primary neurites connected to the MCCl; PC: protocerebrum; VLP: ventro-lateral protocerebrum. Directions: A, anterior; D, dorsal; L, lateral; M, medial; P, posterior; V, ventral. Scale bars = 100 μm. The scale bar in **H1** was also applied to **A1**–**G1** and **A2**–**H2**, and in **H3** applied to **A3**–**G3**.

Six ALs from 3 *H. armigera* females were analyzed and reconstructed. Immunolabeling with anti-synapsin antibody revealed that the AL of the female consists of a large number of glomeruli and a fibre core, called AL hub^36^. All glomeruli are arrayed in a demarcated layer surrounding the hub (Fig. 1C–E). Two large cell clusters surrounding the glomeruli, the medial cell cluster (MCCl) and the lateral cell cluster (LCCl), could be observed (Fig. 1a3). The diameters of the glomerular neuropil area in x, y, and z axis are 262.99 ± 17.52 μm, 276.49 ± 8.79 μm, and 139 ± 5.59 μm (mean ± SD, n = 6), respectively (Table 1). The total volume of glomeruli is 2 555 129 ± 250 411 μm^3^ (mean ± SD, n = 6) (Table 1). Each AL contains 81 glomeruli. The number of glomeruli is the same across individuals and also in the left and right AL within individuals.

### Glomerular identification and clusters in female *H. armigera*

The procedure for identifying and naming the individual female glomeruli is the same as that used for males^35^. Homologous glomeruli in different preparations were given the same name and color. By taking the complete map of the male AL as reference, female glomeruli that showed similar shape, and position were easily identified and thus determined as homologous (Fig. 1). Five female-specific glomeruli and seventy six being homologues to the glomeruli in the male, i.e. G1-G74, G76, and G77, were found in the female AL. G75, which is located dorso-anteriorly to G49 in the male, was not found in the female. Like in the report on the male, each identified glomerulus of the female was categorized into one of four clusters: the female-specific glomerular complex (Fx), posterior complex (PCx), labial-palp pit organ glomerulus (LPOG), and OGs.

At the entrance of antennal nerve into the AL, at the corresponding position of male MGC, five female-specific glomeruli were identified: the central large female glomerulus (cLFG), and F1-F4 (Fig. 1; Figure S1). No homologous glomeruli of cLFG, and F1-F4 were observed in males. The female-specific glomeruli are categorized as the cluster of Fx (Figure S1). In the previous report by Skiri *et al*.^3^ there are 3 female-specific glomeruli, i.e. cLFG and two additional glomeruli. Two female-specific glomeruli, F1 and F4, were newly identified compared to the previous report. Dorso-posteriorly to the Fx, nine glomeruli, i.e. G49-G52, G54-G57, and G63, are positioned together, separated from the remaining glomeruli (Fig. 1c–F; Figure S1). They are categorized as the PCx. Among these glomeruli, G63 is newly identified in the present study. Most ventrally, the largest glomerulus in female AL appears, G38, LPOG, located at the outer layer of glomeruli G27, G28, G39, G64, G65, G68, and G71. It was identified as one distinct category (Fig. 1F,G; Figure S1). The fourth category, OG, includes the remaining 66 glomeruli, i.e. G1-G37, G39-G48, G53, G58-G62, G64-G74, G76, and G77 (Figure S1). The OGs are arranged in a single peripheral layer forming a ball-like surface (Fig. 1G,H; Figure S1). G64-G74, G76, and G77 are newly identified glomeruli, most of which were previously considered as a part of the protocerebrum^30^.

### Glomerular volumes of female *H. armigera*

The volumes of the individual glomeruli varied in a range from 2737 to 103 941 μm^3^, with mean value of 31 545 μm^3^ (Table 1; Figure S2). A large portion of the glomeruli, about 56, shows volumes between 20 000 and 50 000 μm^3^, and there are 25 glomeruli with volumes between 20 000 and 30 000 μm^3^ (Figure S2). About 6 glomeruli have smaller size than 10 000 μm^3^ and about 9 have larger size than 50 000 μm^3^ (Figure S2). The largest glomerulus in the female AL is the LPOG, i.e. G38, with mean value of 82 890 μm^3^, which accounts for 3.24% of the total glomerular volume (Table 2; Figure S2). The total volume of the Fx is 138 689 μm^3^, which accounts for 5.40% of the total glomerular volume, and the average volume of each glomerulus in the Fx is 27 737 μm^3^ (Table 2). The volume of cLFG is the 51 942 μm^3^, about twice times larger than that of the other female-specific glomeruli. The glomeruli of the PCx account for 11.85% of the total glomerular volume (Table 2). Within the PCx, the largest glomerulus is G57 with 47 430 μm^3^, the smallest is G55 with 19 353 μm^3^, and the mean volume of the PCx glomeruli is 32 625 μm^3^ (Figure S2; Table 2). The OG constitutes the largest part of the AL and accounts for 79.79% of the total glomerular volume (Table 2). The mean volume of each OG is 30 907 μm^3^ (Table 2). The smallest ordinary glomeruli include G64-G69, G76, and G77; most of which have volumes below 10 000 μm^3^. The largest ordinary glomeruli include G36, G37, G39, G48, G53, G71, and G72, having volumes exceeding 45 000 μm^3^ (Figure S2).

### Intra- and inter-individual variation of antennal lobe glomeruli

The number of glomeruli in each of the 6 ALs is constant, with no variation. The correlation coefficients regarding glomerular volumes for the left and right ALs of the same brain and across female individuals are significantly different from 0, indicating that the glomeruli of the left and right AL as well as different individuals have in general the similar size (Table 3; Table S1).

Intra- and inter-individual variation of male glomeruli was also examined in the present study, including 8 ALs from 4 male individuals. The number of glomeruli in each AL is previously reported to be quite constant as well^35^. As regards size of homologous glomeruli in the male, the correlation coefficients show that the left and right ALs as well as different individuals have, in general, the similar glomerular volumes (Table 3). By comparing each of the individual glomerulus in the right and the left AL, only G4 from the cluster of OG in male was found to be different (Table S1, indicated by asterisks).

### Sexual dimorphism of antennal lobe glomeruli

As suggested by Rospars and Hildebrand^26^, the sexual dimorphism of AL glomeruli were defined as sexual specificity and sexual dimorphism in a strict sense. Sex-specific glomeruli are presence in one sex but not in the other. Sexual dimorphic glomeruli are presence in both sexes, but show differences in locations or volumes. While that presence in both sexes and have the same locations and volumes are sexual isomorphic glomeruli. Homologous glomeruli between female and male include the sexual dimorphic and isomorphic ones glomeruli.

**Figure 2:**
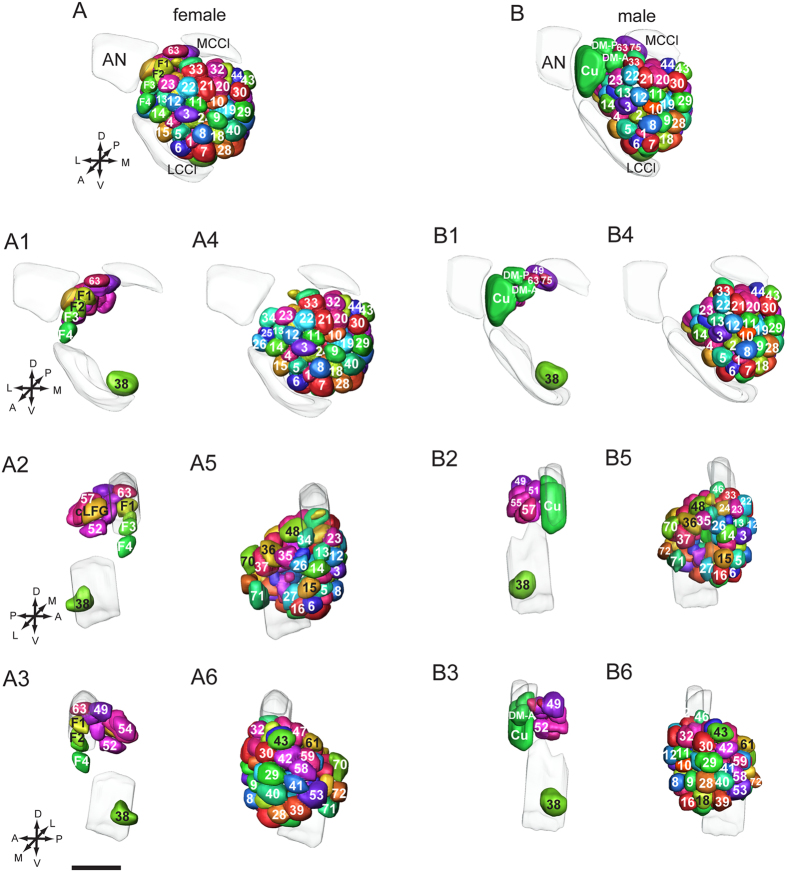
Comparison of the complete glomerular map of female and male in different views. (**A**) Three-dimensional reconstruction of female antennal lobe glomeruli; (**B**) Three-dimensional reconstruction of male antennal lobe glomeruli; **A1–A3**: Three-dimensional reconstruction of the female-specific glomerular complex, posterior complex, and LPOG in different views; **A4–A6**: Three-dimensional reconstruction of ordinary glomeruli of female in different views; **B1–B3**: Three-dimensional reconstruction of the MGC, posterior complex, and LPOG in different views; **B4–B6**: Three-dimensional reconstruction of ordinary glomeruli of male in different views. AN: antennal nerve; cLFG: central large female glomerulus; Cu: cumulus; DM-A: anterior dorso-posterior glomeruli; DM-P: posterior dorso-medial glomeruli; LCCl: lateral cell body cluster; MCCl: medial cell body cluster. Directions: A, anterior; D, dorsal; L, lateral; M, medial; P, posterior; V, ventral. Scale bar = 100 μm. The scale bar in **A3** also applies to **A**, **A1**, **A2**, **A4**–**A6**, B, and **B1**–**B6**.

The complete map of three-dimensional reconstructions, plus the same naming system and color coding for homologous glomeruli facilitate comparisons between the two sexes (Fig. 2; Figure S3). Sex-specific glomeruli were found in both female and male *H. armigera*. Five female-specific glomeruli constitute the Fx including cLFG and F1-F4, while three male-specific glomeruli form the MGC, including Cumulus (Cu), anterior dorso-medial glomerulus (DM-A), and posterior dorso-medial glomerulus (DM-P) (Fig. 2; Figure S3). In addition, G75 which is located dorso-anteriorly to G49 was found in males only. The total volume of the sex-specific glomeruli is also different in males and females; MGC is much larger than the Fx (Table S1). Except for G75, the glomeruli in the clusters of PCx, LPOG, and OG are homologous in shape and location across the two species (Fig. 2; Figure S3). Generally, corresponding glomeruli also show high correlation in size across male and female (Table 3). Volume differences apply only to the PCx glomeruli G49, G51, G52, G54, G55, and G57. The volumes of G52 and G57 are larger in female, while the others are larger in male. Also, the total PCx volume is larger in male (Table S1). The LPOG, i.e. G38, is also larger in male (Table S1). In the cluster of OG, only 13 glomeruli, i.e. G1, G3, G7, G12, G14, G18, G32, G35, G36, G37, G53, G61, and G70 show difference in volume across the two species. The volumes of G1, G7, G14, G35, and G36 are larger in female, while the remaining are larger in male (Table S1). However, the total volume of the ordinary glomeruli is the similar in male and female (Table S1).

## Discussion

### The number of antennal lobe glomeruli in *H. armigera* and other moths

Like in male *H. armigera*, a relatively large number of AL glomeruli were identified in the female. Compared with the previous report of 65 glomeruli in the female of this species^30^, 16 glomeruli, i.e. F1, F4, G63-G74, G76, and G77, are newly identified in the present study. Among of them, the glomeruli G64-G74 are located in the posterior region of the AL. The reason why they were overlooked in the previous study may be due to the difficulty of differentiating them from the protocerebrum^30^,^35^. Besides the newly discovered glomeruli located posteriorly, a group of additional glomeruli including F1, F4, G63, G76, and G77 were also not previously identified. The reason might have been that they are small and located closely to their adjacent glomeruli.

In a number of previous studies, the reported number of glomeruli in the AL of several female moth species is approximately 65, i.e. 65 in *H. armigera*^30^, 66 in *H. assulta*^30^, 66 in *Heliothis virescens*^27^, about 60 in *S. littoroalis*^37^). In the Tortricidae, the reported number of glomeruli shows a larger variation, i.e. 54 in *Cydia molesta*, 49 in *Cydia pomonella*^8^,^33^, and 71 in *Lobesia botrana*^29^. Compared to the number of 81 glomeruli reported in the current study of female *H. armigera*, however, all the formerly reported numbers are quite low. This indicates that there might be some additional glomeruli in the AL of others moth species as well. Recently, the number of glomeruli in female *Manduca sexta*^26^ was updated from 63 to 71^38^. Unfortunately, however, the newly identified glomeruli were not specified.

In addition to the high number of glomeruli, we also found that the number is constant in female *H. armigera*. Different individuals, including both right and left ALs in the same preparation, show the same number of 81 glomeruli. This is also different from previous reports of other moth species^29^,^31^,^32^,^33^. For instance, the counted number of glomeruli in *L. botrana* ranged 60–71^29^, in *S. littoroalis* 55–63^31^, in *Bombyx mori* 58–62^32^, and in *Cydia molesta* 49–53^33^. The reasons for such a variation might be, (1) there are some anomalous glomeruli, or missing glomeruli, in particular individuals of these species. Such glomeruli could thus be found in one individual, but not in another, or could be found in one AL, but not in the contralateral AL; (2) the glomeruli situated posteriorly are difficult to identify because of poorly visualization of the border between the AL and the protocerebrum^26^,^31^,^33^.

### Sexual dimorphism of antennal lobe glomeruli in *H. armigera* and its significance

It is well known the ALs of moths are sexually dimorphic. By comparing the complete map of three-dimensional reconstructions of female and male of *H. armigera*, we found differences in the sex-specific glomeruli as well as in some of the homologous glomeruli.

In the present study, two more female-specific glomeruli than those previously reported were identified^30^. Among the 5 female-specific glomeruli, one is large and four are small (Figure S1; Table S1). The large one is the cLFG. A similar large female-specific glomerulus was also found in *M. sexta, H. virescens*, and *H. assulta*^26^,^27^,^30^. The number of female-specific glomeruli seems to vary in different species, for example, the number is reported to be 4 in *H. assulta*, 5 in *M. sexta*, and 9 in *C. molesta*^30^,^33^,^38^. In contrast to the MGC, the function of female-specific glomeruli is poorly understood. They might be involved in encoding information about odorants mediating female-specific behavior, as for instance, oviposition. The lateral LFG of *M. sexta* has been shown to represent the plant odor linalool, which is important for oviposition behavior of the moth^39^.

Somewhat different from the report on *M. sexta*^26^, the difference between male and female *H. armigera* included not only the sex-specific glomeruli but also a substantial part of the PCx. G75 was found only in the male, not in the female. Futhermore, the volumes of G49, G51, G52, G54, G55, and G57 are different in female and male. The function of the PCx in either gender is not yet known. Tracing of physiologically characterized OSNs in male *Helicoverpa zea* and *Heliothis subflexa* has shown that one neuron being confined to the most abundant male-specific sensillum type, housing the (Z)-11-hexadecenal-sensitive OSN, projects into the G49^40^,^41^. Also, similar results have been found in male *H. assulta* and *H. virescens*^42^,^43^. In addition, the somata of PNs innervating the PCx are located in MCCl, which houses the somata of many MGC-associated PNs^35^,^44^. Taken together, we can assume that PCx might play a role related to that of the sex-specific glomeruli.

LPOG (G38) of *H. armigera* is also sexually dimorphic in volume, which is different from the previous report^30^. The reason for the discrepancy in two studies might be, (1) the low number of preparations investigated in the previous study, and (2) that the boundary of the LPOG is difficult to delineate because it located ventro-posteriorly in the AL. In the previous study on the *H. assulta* male, the LPOG consisted of both G71 and G72, and the volume was therefore larger^27^. Sexually dimorphic LPOGs were also found in *C. molesta*^33^. Here the female LPOG is larger than that of the male, just reversed to what we found in *H. armigera*. It had been demonstrated that the LPOG of *M. sexta* receives input about CO_2_^45^ Alao, in *H. armigera*, the LPOG is shown to be innervated by CO_2_-responding sensory axons from the LPO^46^,^47^. The different volumes of the LPOGs in female and male might indicate that CO_2_ elicit different behaviors in the two sexes.

In the cluster of OG, all the homologous glomeruli in female and male show similar shape and location, and most of them show similar size as well. Only 13 glomeruli, i.e. G1, G3, G7, G12, G14, G18, G32, G35, G36, G37, G53, G61, and G70 show significant differences in volume across the two sexes. Generally, OGs have been reported to process plant odor information^14^, however, the function of the OGs in *H. armigera* has not yet been studied. Attempts to characterize glomeruli physiologically by using electrophysiological recording and staining combined with three-dimensional reconstruction have been conducted in several moths species, including *C. pomonella*^8^, *A. ipsilon*^28^, *L. botrana*^29^, *S. littoralis*^37^, *C. molesta*^48^. In general, the pattern of responses of OG to plant volatiles is broad and complex, including different OGs showing response to the same volatile, and the same OG showing responses to different volatiles. In *C. molesta* and *C. pomonella*, the non-segregated representation of pheromones and plant odours in the AL makes the response pattern even more complex^8^,^48^. By comparing the representations of odours in the AL of *C. pomonella*, different female and male glomeruli respond to the same compound whereas the same compound activates different glomeruli^8^. Whether the sex-dimorphic OGs in *H. armigera* also represent distinct responses patterns to odours across the two sexes is an open question.

## Conclusion

In summary, 81 glomeruli were identified in the female *H. armigera,* of which 16 were newly identified. The number is considerably larger than that previously reported in this species and in others moth species as well. The general anatomical organization of the AL of the female *H. armigera* is similar to that of the male and all female glomeruli were classified into four sub-groups, corresponding to those of the male: (1) the female-specific glomerular complex, (2) the posterior complex, (3) the labial-palp pit organ glomerulus, and (4) the ordinary glomeruli. Such findings may provide a reference to examine the AL organization and to identify more glomeruli in other moth species. A global-wide comparison on the complete glomerular map of the female and male AL of *H. armigera* revealed that sexual dimorphism applies to the sex-specific glomeruli and some homologous glomeruli, which indicate that sex-related olfactory behavior involves odors not yet identified. The findings in the present study may serve as, (1) a reference to examine the AL organization and to identify more glomeruli in other moth species, (2) a map to investigate the function of glomeruli and to understand the neural basis for the divergent olfactory behaviors in female and male.

## Materials and Methods

### Insects

Insects of *Helicoverpa armigera*, which were reared on an artificial diet under the conditions of 16/8 h light/dark circle, 27 °C and 70% relative humidity, were obtained from the laboratory colony. During early pupal stage, females and males were separated and each sex was reared in separated containers. Two to five days after eclosion, *H. armigera* were used for the experiments and the brains were dissected out in fresh Ringer’s solution (in mM: 150 NaCl, 3 CaCl2, 3 KCl, 25 Sucrose, and 10 N-tris (hydroxymethyl)-methyl-2-amino-ethanesulfonic acid, pH 6.9).

### Immunocytochemistry

In order to visualize the AL glomeruli, immunostaining with anti-synpasin antiserum of SYNORF1 (Developmental Studies Hybridoma Bank, University of Iowa, Iowa City) marking presynaptic boutons, was performed, with the same procedure as for the male^35^. After fixation in a 4% paraformaldehyde solution in phosphate-buffered saline (PBS; in mM: 684 NaCl, 13 KCl, 50.7 Na2HPO4, 5 KH2PO4, pH 7.4) for 2 hours at room temperature or overnight at 4 °C, the brains were rinsed in PBS 4 × 15 min. In order to minimize non-specific staining, the brains were pre-incubated in 5% normal goat serum (Sigma, St. Louis, MO) in PBS containing 0.5% Triton X-100 (PBSX; 0.1 M, pH 7.4) for 3 hours at room temperature. Following the incubation in the primary antibody, SYNORF1, at 1:100 in PBSX at 4 °C for 5 days, the brains were rinsed in PBS 6 × 20 min. Then the brains were incubated in the secondary antibody, Cy2-conjugated anti-mouse (Invitrogen, Eugene, OR; dilution 1:300 in PBSX), at 4 °C for 3 days, before washing 6 × 20 min in PBS, dehydrating with ascending ethanol series, and mounting in methylsalicylate.

### Confocal image acquisition and digital 3D-reconstruction

The AL were scanned by using a confocal laser scanning microscope (LSM 510, META Zeiss, Jena, Germany) with objectives of 20 × (Plan-Neofluar 20 × /0.5l) at the step size of 3 μm to obtain the serial optical images. A 488-nm line of an Argon laser was used to excite the Cy2. The resolutions of the confocal images are 1024 × 1024 pixels.

In order to visualize the three-dimensional structure of the AL glomeruli, the confocal image stacks were subject to reconstruction by using the AMIRA software (AMIRA 5.3, Visage Imaging, Fürth, Germany). The glomeruli were labeled as previously described for male *H. armigera* by using the segmentation editor, including the “Brush” and “Interpolate” tools^35^. The volumes of the individual glomeruli were measured by means of the “TissueStatistics” tool and the quantitative data were imported to Excel for further processing.

### Glomerular nomenclature

The glomeruli were identified and named according to the report for male *H. armigera*^34^. Like in the male, all female glomeruli were classified into four clusters: the female-specific glomerular complex (Fx), the posterior complex (PCx), the ordinary glomeruli (OGs), and the Labial-palp pit organ glomerulus (LPOG). The five glomeruli of the Fx were named the central large female-specific glomeruli (cLFG), F1, F2, F3, and F4, respectively. The remaining glomeruli were numbered from G1 in a clockwise direction for the left AL (and anti-clockwise direction for the right), from the anterior to the posterior part of the AL. The orientation refers to the axis of the insect body. The terms right and left are used from the insect’s point of view.

### Statistics

The volumes of the AL and each individual glomerulus were calculated in the office software of Excel, and are presented as mean ± SD. In order to examine the volume differences of homologous glomeruli between the left and right AL (intra-individual), between individuals (inter-individual), and between sexes (inter-sexual), the paired *t* tests were performed. A non-parametric analysis with Mann-Whitney U test was also performed to examine the volume differences of the homologous glomeruli between the left and right AL and between the sexes. The software of SPSS (IBM, SPSS, Statistics, version 21) was used for the statistical tests.

## Additional Information

**How to cite this article**: Zhao, X.-C. *et al*. A global-wide search for sexual dimorphism of glomeruli in the antennal lobe of female and male *Helicoverpa armigera. Sci. Rep.* **6**, 35204; doi: 10.1038/srep35204 (2016).

## Supplementary Material

Supplementary Information

## Figures and Tables

**Table 1:** Volumes and number of glomeruli in the antennal lobe of female *Helicoverpa armigera.*

Preparation	Diameter (μm)			Volume of all glomeruli (10^3^ μm^3^)	Number of glomeruli	Largest glomerulus (10^3^ μm^3^)	Smallest glomerulus (10^3^ μm^3^)	Mean volume of glomeruli (±SD) (10^3^ μm^3^)
	X-axis	Y-axis	Z-axis					
Female1 R	262.86	267.24	138	2584.85	81	78.79	2.74	31.91 ± 14.33
Female1 L	261.99	277.78	144	2319.73	81	81.69	3.96	28.64 ± 13.56
Female2 R	240.82	266.31	138	2418.93	81	65.19	4.40	29.86 ± 13.53
Female2 L	255.91	290.04	132	2319.17	81	81.09	3.12	28.63 ± 13.59
Female3 R	294.46	279.63	135	2933.64	81	103.94	4.82	36.22 ± 17.97
Female3 L	261.91	277.93	147	2754.56	81	94.98	4.82	34.01 ± 15.14
Mean (±SD)	262.99 ± 17.52	276.49 ± 8.79	139 ± 5.59	2555.13 ± 250.41	81	—	—	31.65 ± 13.38

R and L indicate the right and left antennal lobe respectively.

**Table 2:** Clusters of glomeruli in the antennal lobe of female *Helicoverpa armigera.*

Cluster	Volume (10^3^ μm^3^)	Relative size according to all glomeruli (%)	Number of glomeruli	Average volume of each glomerulus (10^3^ μm^3^)
Fx	138.69 ± 25.60 (6)	5.40 ± 0.54 (6)	5 (6)	27.74 ± 14.93
PCx	293.63 ± 20.56 (6)	11.58 ± 1.30 (6)	9 (6)	32.63 ± 9.62
OG	2039.92 ± 21.81 (6)	79.79 ± 1.23 (6)	66 (6)	30.91 ± 12.40
LPOG	82.89 ± 14.58 (6)	3.24 ± 0.41 (6)	1 (6)	—
All	2555.13 ± 250.41 (6)	—	81 (6)	31.54 ± 13.40

The data of volume and relative size are presented as Mean ± SD. Fx: female-specific glomerular complex; LPOG: labial-pit organ glomerulus; OG: ordinary glomeruli; PCx: the posterior complex.

**Table 3:** Coefficient of correlation (*r*) of glomeruli size within, between individuals and sexes.

Comparison		Number of glomeruli paired	Size *r*	*P-*value	Paired *t* test		
					*t-*value	*df*	*P-*value
Intra-individual Female	Female1 R/L	81	0.894	<0.001	4.558	80	0.000
	Female2 R/L	81	0.862	<0.001	1.558	80	0.123
	Female3 R/L	81	0.731	<0.001	1.603	80	0.113
Inter-individual Female	Female1/Female2	81	0.862	<0.001	1.318	80	0.191
	Female1/Ffemale3	81	0.891	<0.001	6.229	80	0.000
	Female2/Female3	81	0.855	<0.001	6.609	80	0.000
Intra-individual Male	Male1 R/L	79	0.969	<0.001	1.033	78	0.305
	Male2 R/L	79	0.967	<0.001	0.726	78	0.470
	Male3 R/L	80	0.963	<0.001	0.291	79	0.770
	Male4 R/L	79	0.942	<0.001	0.761	78	0.449
Inter-individual Male	Male1/Male2	79	0.952	<0.001	2.704	78	0.008
	Male1/Male3	79	0.951	<0.001	1.237	78	0.220
	Male1/Male4	79	0.963	<0.001	1.359	78	0.178
	Male2/Male3	79	0.950	<0.001	3.666	78	0.000
	Male2/Male4	79	0.954	<0.001	2.632	78	0.010
	Male3/Male4	79	0.962	<0.001	1.804	78	0.075
Inter-sex (without Sex-specific glomeruli)	Male1/Female1	75	0.817	<0.001	3.330		0.001
	Male1/Female2	75	0.809	<0.001	4.182	74	0.000
	Male1/Female3	75	0.831	<0.001	0.747	74	0.459
	Male2/Female1	75	0.777	<0.001	4.266	74	0.000
	Male2/Female2	75	0.815	<0.001	5.345	74	0.000
	Male2/Female3	75	0.794	<0.001	1.084	74	0.282
	Male3/Female1	76	0.798	<0.001	2.122	75	0.037
	Male3/Female2	76	0.832	<0.001	3.192	75	0.002
	Male3/Female3	76	0.832	<0.001	1.810	75	0.074
	Male4/Female1	75	0.793	<0.001	3.308	74	0.001
	Male4/Female2	75	0.813	<0.001	4.215	74	0.000
	Male4/Female3	75	0.818	<0.001	0.032	74	0.974

Male1-Male4, four male individuals. Female1-Female3, three female individuals.
