# Supplementary material for: A global-wide search for sexual dimorphism of glomeruli in the antennal lobe of female and male Helicoverpa armigera
Source: Sci Rep. 2016 Oct 11;6:35204. doi: 10.1038/srep35204 (PMC5057091; doi:10.1038/srep35204)
Supplement: Supplementary Information [file srep35204-s1.doc]

**Supplementary information**

**A global-wide search for sexual dimorphism of glomeruli in the antennal lobe of female and male *Helicoverpa armigera***

Xin-Cheng Zhao^1^*, Bai-Wei Ma^1^, Bente G. Berg^2^, Gui-Ying Xie^1^, Qing-Bo Tang^1^, Xian-Ru Guo^1,3^

1 Department of Entomology, College of Plant Protection, Henan Agricultural University, Zhengzhou, 450002, China

2 Department of Psychology, Norwegian University of Science and Technology, Trondheim 7489, Norway

3 Collaborative Innovation Center of Henan Grain Crops, Zhengzhou, 450002, China

* corresponding author:

Dr. Xin-Cheng Zhao, Department of Entomology, College of Plant Protection, Henan Agricultural University, Zhengzhou, Henan 450002 China. Tel: 0086 371 6355 8170. Email: [xincheng@henau.edu.cn](mailto:xincheng@henau.edu.cn)

**Supplementary figure legends**

**Figure S1**

Three-dimensional reconstruction of the antennal lobe (AL) glomeruli including the sub-groups they form. **A1-F1:** Three-dimensional reconstruction of the four glomerular clusters in different views, Including the female-specific glomerular complex (Fx), the labial-pit organ glomerulus (LPOG), the posterior complex (PCx), and the ordinary glomeruli (OG). **A2-F2:** Three-dimensional reconstruction of the Fx, the LPOG, and the PCx in different views. **A3-F3:** Three-dimensional reconstruction of the OG in different views. Frontal view (A1-A3). Posterior view (B1-B3). Dorsal view (C1-C3). Ventral view (D1-D3). Lateral view (E1-E3). Medial view (F1-F3). cLFG: central large female glomerulus. Directions: A, anterior; D, dorsal; L, lateral; M, medial; P, posterior; V, ventral. Scale bar = 100 µm. The scale bar in F1 also applies to A1-E1, A2-F2, and A3-F3.

**Figure S2**

Distribution of glomerular size. **A:** Histograms showing glomerular size, from the smallest to the largest. **B:** Histograms demonstrating number of glomeruli possessing distinct volumes.

**Figure S3**

Comparison of individual glomeruli in female and male. The hidden glomeruli in the posterior part of the AL are shown by removing the anterior ones. **A1-A4**: Individual glomeruli of the Fx, PCx, and G38 of female; **B1-B4**: Individual glomeruli of the MGC, PCx, and G38 of male; **C1-C5**: Individual glomeruli of OG of female; **D1-D5**: Individual glomeruli of OG of male. AN: antennal nerve; cLFG: central large female glomerulus; Cu: cumulus; DM-A: anterior dorso-posterior glomeruli; DM-P: posterior dorso-medial glomeruli; LCCl: lateral cell body cluster; MCCl: medial cell body cluster. Directions: A, anterior; D, dorsal; L, lateral; M, medial; P, posterior; V, ventral. Scale bar = 100 µm. The scale bar in A4 also applies to A1- A3, B1-B4, C1-C5, and D1-D5.

**Figure S1**


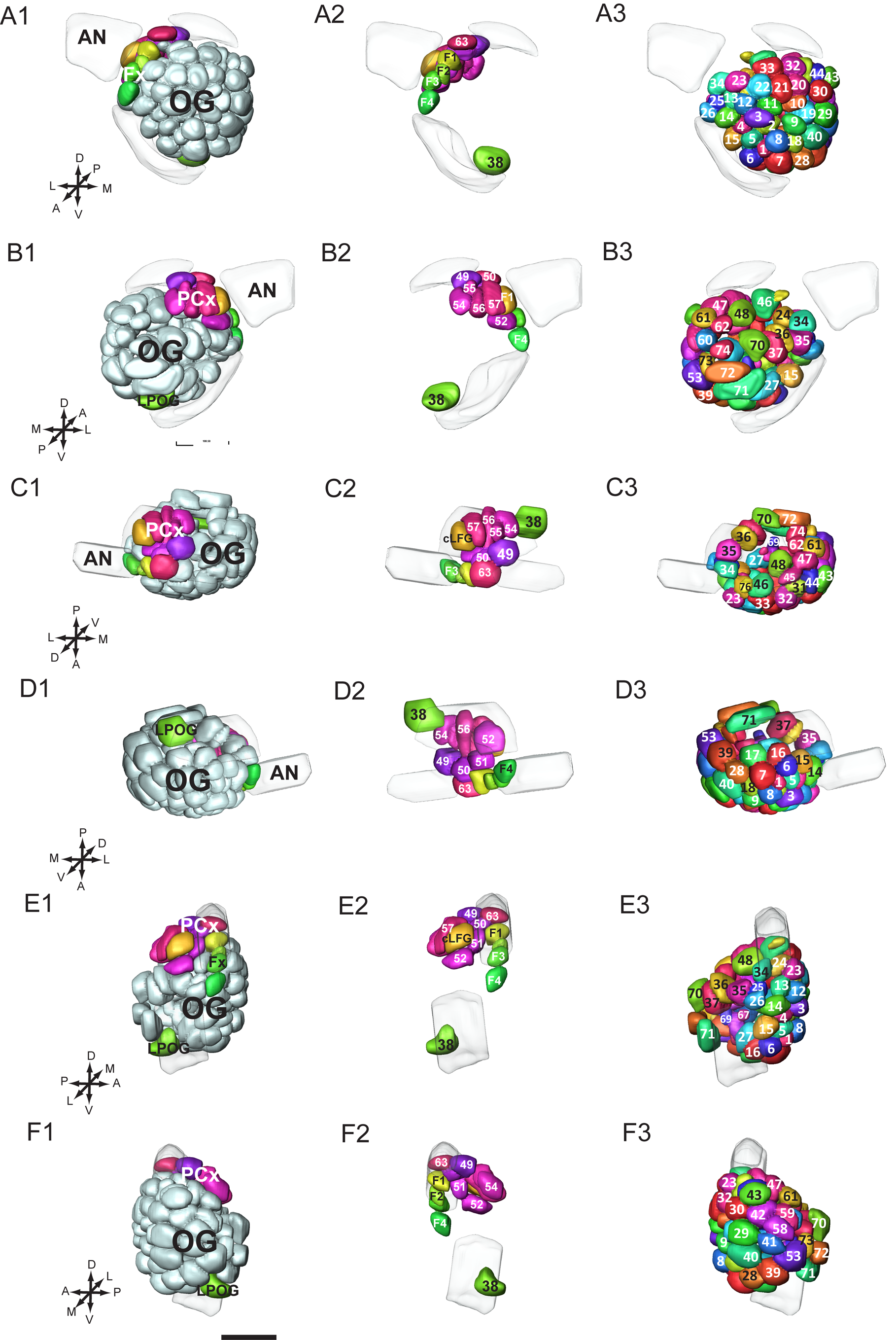


**Figure S2**


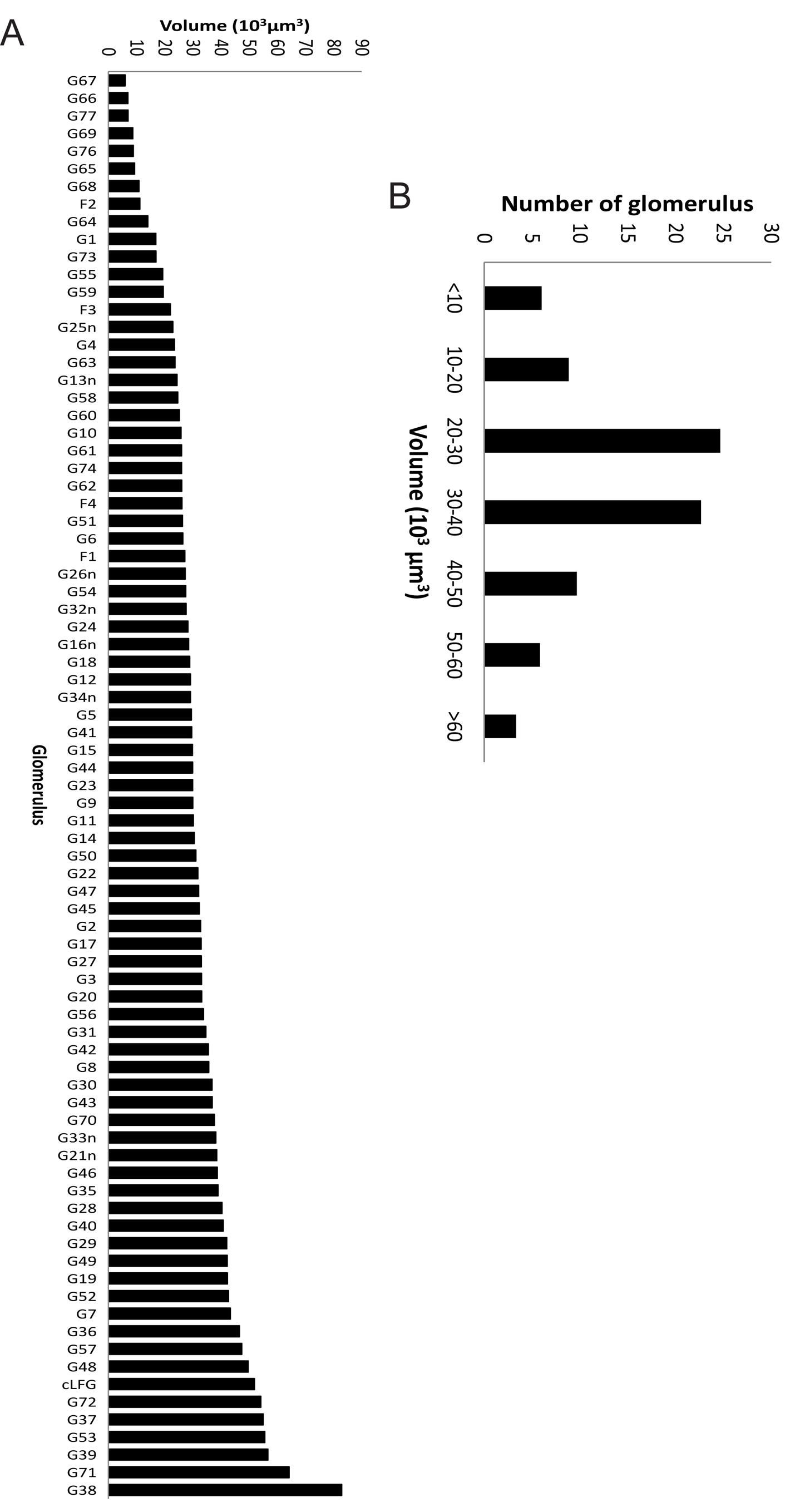


**Figure S3**


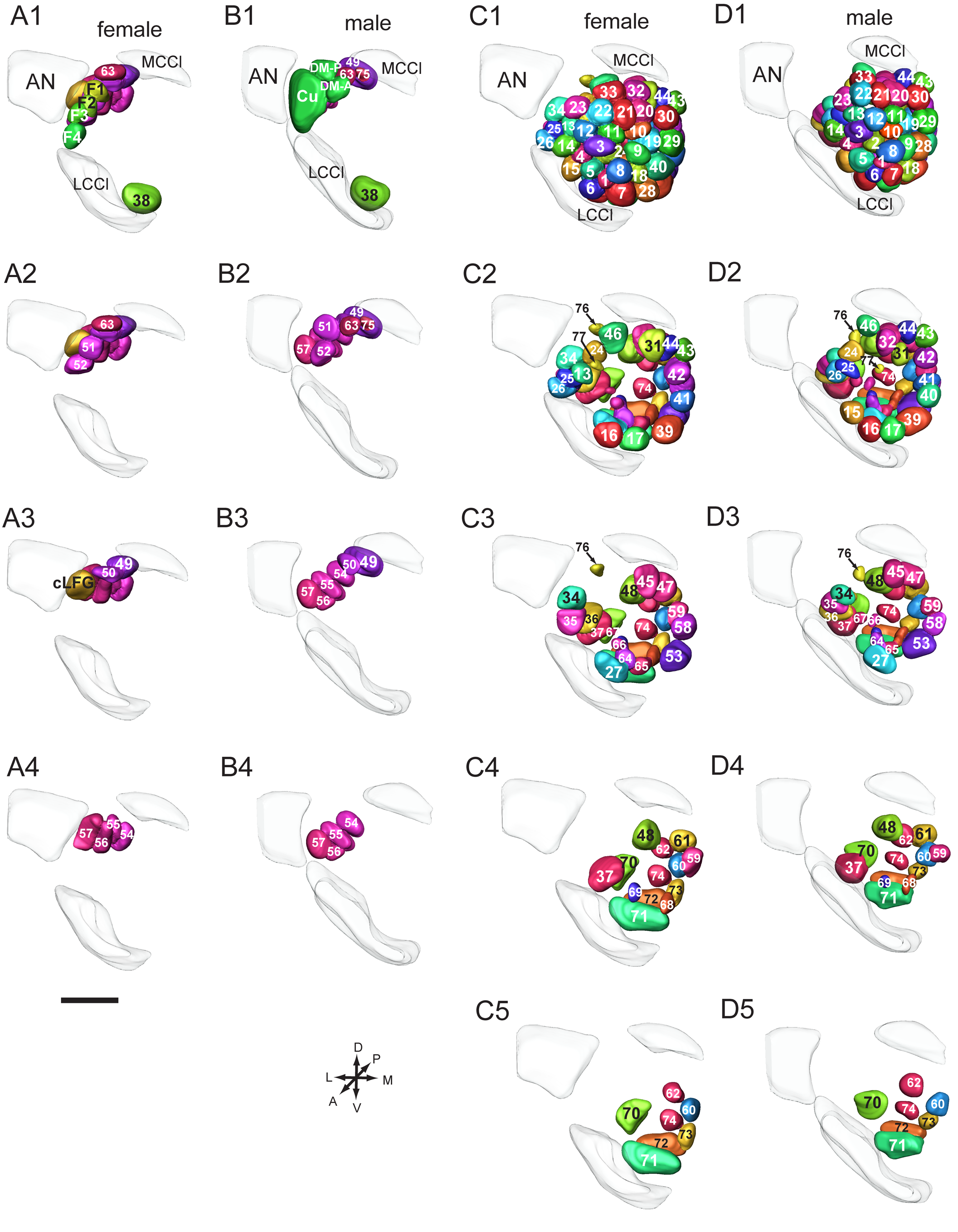


**Table S1 The quantitative comparison of individual glomeruli of female and male.**

| Glomeruli | Female |  |  | Male |  |  | U test for right and left AL of female |  | U test for right and left AL of male |  | U test for female and Male |
| --- | --- | --- | --- | --- | --- | --- | --- | --- | --- | --- | --- |
|  | Volume (10^3^ µm^3^) | n |  | Volume (10^3^ µm^3^) | n |  | *P*-value |  | *P*-value |  | *P*-value |
| Sex-specific glomeruli |  |  |  |  |  |  |  |  |  |  |  |
| cLFG | 51.94 ± 11.31 | 6 |  | - | - |  | 1.000 |  | - |  | - |
| F1 | 27.23 ± 4.90 | 6 |  | - | - |  | 0.700 |  | - |  | - |
| F2 | 11.26 ± 5.55 | 6 |  | - | - |  | 0.700 |  | - |  | - |
| F3 | 22.08 ± 5.69 | 6 |  | - | - |  | 1.000 |  | - |  | - |
| F4 | 26.19 ± 3.38 | 6 |  | - | - |  | 0.700 |  | - |  | - |
| Cu | - | - |  | 222.47 ± 20.07 | 8 |  | - |  |  |  | - |
| DM-A | - | - |  | 45.10 ± 4.97 | 8 |  | - |  |  |  | - |
| DM-P | - | - |  | 46.54 ± 6.22 | 8 |  | - |  |  |  | - |
| OG |  |  |  |  |  |  |  |  |  |  |  |
| G1 | 16.90 ± 1.66 | 6 |  | 14.04 ± 2.51 | 8 |  | 0.100 |  | 0.686 |  | 0.029* |
| G2 | 32.83 ± 7.99 | 6 |  | 33.96 ± 3.17 | 8 |  | 0.400 |  | 1.000 |  | 0.491 |
| G3 | 33.13 ± 9.26 | 6 |  | 40.48 ± 3.22 | 8 |  | 0.100 |  | 0.686 |  | 0.043* |
| G4 | 23.56 ± 6.74 | 6 |  | 23.23 ± 2.58 | 8 |  | 0.700 |  | 0.029* |  | 0.852 |
| G5 | 29.49 ± 7.35 | 6 |  | 30.49 ± 5.90 | 8 |  | 0.700 |  | 0.486 |  | 0.662 |
| G6 | 26.53 ± 11.01 | 6 |  | 21.02 ± 4.45 | 8 |  | 1.000 |  | 0.686 |  | 0.491 |
| G7 | 43.38 ± 6.97 | 6 |  | 34.48 ± 6.84 | 8 |  | 0.400 |  | 0.343 |  | 0.043* |
| G8 | 35.71 ± 7.82 | 6 |  | 33.99 ± 5.08 | 8 |  | 0.100 |  | 0.686 |  | 0.852 |
| G9 | 30.06 ± 7.66 | 6 |  | 31.05 ± 2.58 | 8 |  | 0.400 |  | 0.343 |  | 0.852 |
| G10 | 25.88 ± 5.21 | 6 |  | 28.41 ± 4.02 | 8 |  | 0.400 |  | 1.000 |  | 0.181 |
| G11 | 30.22 ± 9.29 | 6 |  | 27.55 ± 6.13 | 8 |  | 0.400 |  | 1.000 |  | 0.950 |
| G12 | 29.19 ± 6.32 | 6 |  | 34.92 ± 4.79 | 8 |  | 0.700 |  | 0.486 |  | 0.043* |
| G13 | 24.44 ± 6.81 | 6 |  | 28.11 ± 3.44 | 8 |  | 1.000 |  | 1.000 |  | 0.228 |
| G14 | 30.53 ± 3.36 | 6 |  | 24.46 ± 5.12 | 8 |  | 0.700 |  | 0.343 |  | 0.029* |
| G15 | 29.93 ± 3.42 | 6 |  | 30.75 ± 5.76 | 8 |  | 0.200 |  | 0.686 |  | 0.755 |
| G16 | 28.53 ± 4.01 | 6 |  | 31.51 ± 6.46 | 8 |  | 1.000 |  | 0.486 |  | 0.345 |
| G17 | 33.00 ± 3.47 | 6 |  | 33.38 ± 6.58 | 8 |  | 0.400 |  | 0.686 |  | 0.491 |
| G18 | 28.94 ± 3.19 | 6 |  | 34.29 ± 2.32 | 8 |  | 0.700 |  | 1.000 |  | 0.005* |
| G19 | 42.36 ± 10.67 | 6 |  | 38.13 ± 7.66 | 8 |  | 0.700 |  | 0.343 |  | 0.228 |
| G20 | 33.21 ± 7.29 | 6 |  | 38.36 ± 5.52 | 8 |  | 0.100 |  | 0.686 |  | 0.191 |
| G21 | 38.55 ± 10.21 | 6 |  | 34.91 ± 5.87 | 8 |  | 1.000 |  | 0.886 |  | 0.852 |
| G22 | 31.86 ± 1.23 | 6 |  | 35.70 ± 8.71 | 8 |  | 0.700 |  | 0.686 |  | 0.262 |
| G23 | 30.00 ± 9.24 | 6 |  | 29.44 ± 4.25 | 8 |  | 0.700 |  | 0.886 |  | 0.662 |
| G24 | 28.32 ± 11.69 | 6 |  | 30.13 ± 4.25 | 8 |  | 0.200 |  | 1.000 |  | 0.081 |
| G25 | 22.97 ± 4.98 | 6 |  | 18.63 ± 3.39 | 8 |  | 1.000 |  | 1.000 |  | 0.059 |
| G26 | 27.31 ± 8.42 | 6 |  | 20.96 ± 4.71 | 8 |  | 0.400 |  | 0.686 |  | 0.108 |
| G27 | 33.06 ± 5.83 | 6 |  | 34.31 ± 7.42 | 8 |  | 1.000 |  | 0.686 |  | 0.573 |
| G28 | 40.40 ± 13.35 | 6 |  | 52.18 ± 12.65 | 8 |  | 0.400 |  | 0.114 |  | 0.181 |
| G29 | 42.11 ± 15.12 | 6 |  | 45.26 ± 5.46 | 8 |  | 0.400 |  | 1.000 |  | 0.414 |
| G30 | 36.88 ± 7.36 | 6 |  | 40.72 ± 5.79 | 8 |  | 1.000 |  | 0.486 |  | 0.282 |
| G31 | 34.69 ± 12.04 | 6 |  | 27.32 ± 1.75 | 8 |  | 0.100 |  | 0.200 |  | 0.108 |
| G32 | 27.67 ± 2.55 | 6 |  | 31.96 ± 4.19 | 8 |  | 0.700 |  | 0.114 |  | 0.043* |
| G33 | 38.21 ± 5.32 | 6 |  | 41.04 ± 4.95 | 8 |  | 0.700 |  | 1.000 |  | 0.282 |
| G34 | 29.22 ± 6.67 | 6 |  | 26.07 ± 4.29 | 8 |  | 0.700 |  | 1.000 |  | 0.414 |
| G35 | 39.02 ± 7.28 | 6 |  | 29.19 ± 4.89 | 8 |  | 1.000 |  | 0.686 |  | 0.009* |
| G36 | 46.62 ± 5.12 | 6 |  | 62.03 ± 8.12 | 8 |  | 0.400 |  | 0.686 |  | 0.001* |
| G37 | 55.01 ± 7.15 | 6 |  | 68.78 ± 12.48 | 8 |  | 0.400 |  | 1.000 |  | 0.020* |
| G39 | 56.69 ± 9.01 | 6 |  | 66.50 ± 9.41 | 8 |  | 0.700 |  | 0.343 |  | 0.108 |
| G40 | 40.85 ± 11.73 | 6 |  | 41.45 ± 13.15 | 8 |  | 0.400 |  | 0.686 |  | 1.000 |
| G41 | 29.66 ± 8.41 | 6 |  | 37.88 ± 11.95 | 8 |  | 0.400 |  | 1.000 |  | 0.282 |
| G42 | 35.58 ± 6.07 | 6 |  | 38.36 ± 4.16 | 8 |  | 1.000 |  | 0.686 |  | 0.491 |
| G43 | 36.90 ± 10.91 | 6 |  | 38.27 ± 5.99 | 8 |  | 0.700 |  | 1.000 |  | 0.491 |
| G44 | 29.95 ± 6.10 | 6 |  | 34.91 ± 5.86 | 8 |  | 1.000 |  | 0.486 |  | 0.282 |
| G45 | 32.38 ± 10.51 | 6 |  | 40.27 ± 5.72 | 8 |  | 1.000 |  | 0.486 |  | 0.108 |
| G46 | 38.72 ± 7.81 | 6 |  | 32.70 ± 6.29 | 8 |  | 0.700 |  | 1.000 |  | 0.142 |
| G47 | 32.11 ± 6.30 | 6 |  | 39.17 ± 7.35 | 8 |  | 0.700 |  | 0.886 |  | 0.142 |
| G48 | 49.68 ± 3.33 | 6 |  | 53.41 ± 5.13 | 8 |  | 1.000 |  | 0.114 |  | 0.662 |
| G53 | 55.60 ± 7.50 | 6 |  | 71.79 ± 10.76 | 8 |  | 0.100 |  | 0.486 |  | 0.005* |
| G58 | 24.70 ± 8.15 | 6 |  | 29.96 ± 8.20 | 8 |  | 0.400 |  | 0.886 |  | 0.282 |
| G59 | 19.56 ± 4.39 | 6 |  | 24.22 ± 4.22 | 8 |  | 0.200 |  | 1.000 |  | 0.081 |
| G60 | 25.31 ± 9.94 | 6 |  | 34.52 ± 9.14 | 8 |  | 1.000 |  | 0.114 |  | 0.142 |
| G61 | 26.026 ± 7.63 | 6 |  | 38.23 ± 6.08 | 8 |  | 1.000 |  | 1.000 |  | 0.008* |
| G62 | 26.12 ± 4.60 | 6 |  | 34.85 ± 8.10 | 8 |  | 0.700 |  | 1.000 |  | 0.081 |
| G64 | 14.08 ± 4.89 | 6 |  | 13.22 ± 2.87 | 8 |  | 0.700 |  | 1.000 |  | 1.000 |
| G65 | 9.39 ± 2.92 | 6 |  | 14.59 ± 7.03 | 8 |  | 0.700 |  | 0.343 |  | 0.282 |
| G66 | 7.00 ± 2.41 | 6 |  | 7.64 ± 2.42 | 8 |  | 0.200 |  | 0.486 |  | 0.662 |
| G67 | 6.05 ± 3.41 | 6 |  | 6.29 ± 2.81 | 8 |  | 1.000 |  | 0.486 |  | 0.662 |
| G68 | 10.91 ± 3.60 | 6 |  | 12.07 ± 3.00 | 8 |  | 0.400 |  | 1.000 |  | 0.755 |
| G69 | 8.76 ± 3.38 | 6 |  | 6.89 ± 3.70 | 8 |  | 1.000 |  | 0.686 |  | 0.282 |
| G70 | 37.70 ± 3.81 | 6 |  | 48.42 ± 11.74 | 8 |  | 0.700 |  | 0.686 |  | 0.020* |
| G71 | 64.26 ± 8.94 | 6 |  | 63.46 ± 12.30 | 8 |  | 0.700 |  | 0.886 |  | 0.662 |
| G72 | 54.21 ± 14.68 | 6 |  | 64.16 ± 16.77 | 8 |  | 0.100 |  | 0.886 |  | 0.228 |
| G73 | 16.95 ± 2.16 | 6 |  | 18.13 ± 2.37 | 8 |  | 1.000 |  | 0.343 |  | 0.491 |
| G74 | 26.08 ± 3.50 | 6 |  | 27.86 ± 8.42 | 8 |  | 0.200 |  | 0.343 |  | 0.573 |
| G76 | 7.97 ± 2.16 | 6 |  | 6.12 ± 1.09 | 8 |  | 0.700 |  | 0.486 |  | 0.108 |
| G77 | 7.02 ± 2.35 | 6 |  | 9.71 ± 0.36 | 2 |  | 1.000 |  | 1.000 |  | 0.286 |
| PCx |  |  |  |  |  |  |  |  |  |  |  |
| G49 | 42.25 ± 3.82 | 6 |  | 87.73 ± 9.22 | 8 |  | 0.700 |  | 0.886 |  | 0.001* |
| G50 | 30.35 ± 8.92 | 6 |  | 27.99 ± 12.14 | 8 |  | 0.700 |  | 1.000 |  | 0.491 |
| G51 | 26.38 ± 2.29 | 6 |  | 32.22 ± 4.14 | 8 |  | 0.700 |  | 0.886 |  | 0.020* |
| G52 | 42.75 ± 5.18 | 6 |  | 31.17 ± 7.05 | 8 |  | 0.700 |  | 0.343 |  | 0.005* |
| G54 | 27.51 ± 5.28 | 6 |  | 41.46 ± 9.59 | 8 |  | 0.700 |  | 0.486 |  | 0.013* |
| G55 | 19.35 ± 4.42 | 6 |  | 35.44 ± 12.14 | 8 |  | 1.000 |  | 0.686 |  | 0.005* |
| G56 | 33.85 ± 7.39 | 6 |  | 28.88 ± 7.32 | 8 |  | 0.400 |  | 0.686 |  | 0.181 |
| G57 | 47.43 ± 8.10 | 6 |  | 37.95 ± 5.63 | 8 |  | 1.000 |  | 0.343 |  | 0.020* |
| G63 | 23.76 ± 7.95 | 6 |  | 19.63 ± 3.26 | 8 |  | 0.700 |  | 0.686 |  | 0.662 |
| G75 | - |  |  | 9.73 ± 3.71 | 8 |  | - |  | 0.486 |  | - |
| LPOG |  |  |  |  |  |  |  |  |  |  |  |
| G38 | 82.89 ± 14.58 | 6 |  | 109.02 ± 12.68 | 8 |  | 0.700 |  | 1.000 |  | 0.005* |
|  |  |  |  |  |  |  |  |  |  |  |  |
| Sum of sex-specific | 138.69 ± 25.60 | 6 |  | 314.11 ± 29.39 | 8 |  | 1.000 |  | 0.686 |  | 0.001* |
| Sum of OG | 2039.92 ± 218.10 | 6 |  | 2189.04 ± 60.53 | 8 |  | 0.400 |  | 1.000 |  | 0.108 |
| Sum of PCx | 293.63 ± 20.56 | 6 |  | 352.18 ± 49.92 | 8 |  | 1.000 |  | 0.486 |  | 0.008* |
| Sum of All glomeruli | 2555.13 ± 250.41 | 6 |  | 2964.35 ± 131.02 | 8 |  | 0.400 |  | 0.886 |  | 0.008* |

The data of volume and relative size are presented as Mean ± SD. The asterisk indicates that the data are significantly different at the level of *P* = 0.05. cLFG: central large female glomerulus; Cu: cumulus; DM-A: anterior dorso-posterior glomeruli; DM-P: posterior dorso-medial glomeruli; LPOG: labial-palp pit organ glomerulus; OG: ordinary glomeruli; PCx, posterior complex.
